# The Effect of the Addition of Blue Honeysuckle Berry Juice to Apple Juice on the Selected Quality Characteristics, Anthocyanin Stability, and Antioxidant Properties

**DOI:** 10.3390/biom9110744

**Published:** 2019-11-17

**Authors:** Anna Grobelna, Stanisław Kalisz, Marek Kieliszek

**Affiliations:** 1 Department of Food Technology and Assessment, Institute of Food Sciences, Warsaw University of Life Sciences—SGGW, Nowoursynowska 159 C, 02-776 Warsaw, Poland; anna_grobelna@sggw.pl; 2 Department of Food Biotechnology and Microbiology, Institute of Food Sciences, Warsaw University of Life Sciences—SGGW, Nowoursynowska 159 C, 02-776 Warsaw, Poland

**Keywords:** blue honeysuckle berry, apple, anthocyanins, polyphenols, antioxidant, juice

## Abstract

Apple juice is rich in phenolic compounds that are important as natural antioxidants. In turn, blue honeysuckle berry juice is a valuable source of bioactive ingredients and can be an interesting and beneficial supplement to fruit juices. The aim of this study was to examine the physicochemical and sensory properties of the newly designed mixture of apple juice and blue honeysuckle berry juice. The addition of blue honeysuckle berry juice to apple juice had a significant effect on the content of anthocyanin and vitamin C in the newly designed fruit juices. After production, the content of anthocyanins and polyphenols in the blue honeysuckle berry juice was high (595.39 and 767.88 mg/100 mL, respectively). As the concentration of blue honeysuckle berry juice added to apple juice was increased, the polyphenol content also increased. The juices analyzed after 4 months of storage were lighter and showed a less intense red color than the juices analyzed directly after production. Antioxidant activity (ABTS assay) in the apple juice mixed with 10% blueberry juice was almost 3 times higher than the pure apple juice after 3 months of storage; the addition of 30% blueberry juice significantly increased the antioxidant activity of the apple juice. Thus, the results of this research have expanded the existing knowledge about the health and sensory properties of apple juice mixed with blue honeysuckle berry juice. These findings can be utilized in further research aiming at the development of new products that can meet consumer expectations.

## 1. Introduction

Fruit juices are important products for consumers looking for an alternative to fresh fruit. These are widely consumed by most people because of their freshness, sensory properties, and nutritional value. Increased expectations and increased consumer awareness of the modern methods of processing fruits and vegetables have resulted in the continuous development of the fresh juice industry. The contemporary food industry could not be developed without the production of new products that can exhibit health-promoting effects in addition to reducing the risk of certain diseases. Consumers value those raw materials that are known and liked by them, but are increasingly aware of the impact on health by foods produced using new and less used raw materials.

Apple juice is one of the most popular products in the world, both in terms of the production process and international exchange. Apples play a very important role in the food industry and are one of the fruits widely grown around the world. The largest apple producers in the world include, among others, China, the United States of America (USA), Poland, and Turkey [1]. Currently, several thousand varieties of apples are grown. Old, almost forgotten varieties of apples, which are characterized by a juicy, delicate, and sweet and sour pulp, are becoming more popular. Apple has a pleasant taste and is rich in the gelling agent pectin, which prevents the phase separation of juices [2]. These fruits are also rich in nutrients that have a positive effect on the human body. In addition, apples are a natural and rich source of compounds with antioxidant properties (phenols). However, it should be noted that the content of phenolic compounds in apples is highly dependent on their variety and cultivation practices [3]. These fruits also possess minerals (potassium, magnesium, iron, calcium), quercetin, luteolin, apigenin, ursolic acid, and fiber [4,5]. Moreover, apple juices attract consumers with their sensory properties and can thus be an important part of a menu. It is worth emphasizing that the high-quality apple juice concentrate produced in Poland is more popular among foreign customers. The main advantage of this concentrate is its acidity, the level of which is determined by the variety and climate during the production of fruits.

**Figure 1:**
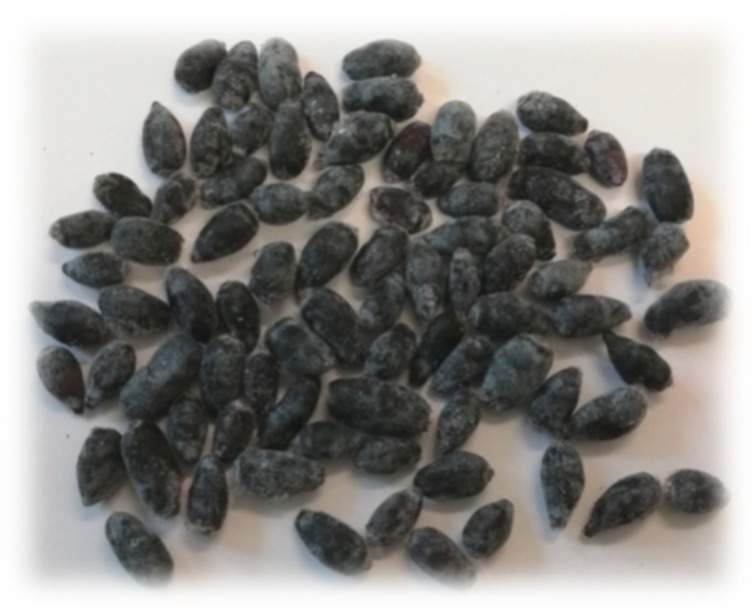
Fruit of blue honeysuckle berry.

In the era of modern globalization, the pursuit of design and production of new food products and their usefulness in processing have resulted in an increase in consumer interest in mixed juices obtained from various fruits. Growing production and consumption of juices incline us to look for ways to make them more attractive sensorially and nutritionally by including other raw materials, with blue honeysuckle berry (*Lonicera caerulea* L.) being one such component. Blue honeysuckle berry is also commonly known as scotch, blue scrub, or haskap. *Lonicera caerulea* L. is a perennial fruit plant belonging to the family Caprifoliaceae (Figure 1) [6,7].

Its fruits are a valuable, natural source of vitamin C (in the level ranging from 29 to 187 mg/100 g) and anthocyanins (including cyanidin-3-*O*-glucoside). B-group vitamins are also found in smaller amounts in their berries [8]. Due to the beneficial chemical composition and attractive aroma, these berries have been used in the food-processing industry as a valuable addition to juices and purees. Undoubtedly, blue honeysuckle berries have greater health-promoting properties than the other commonly consumed berries. The presence of anthocyanins in blue honeysuckle berries contributes to their antioxidant effects [9]. It is believed that blue honeysuckle berry was used in folk medicine to reduce the risk of hypertension, glaucoma, anemia, osteoporosis [10,11], and gastrointestinal disorders [6], and in the treatment of various eye diseases [8]. The current scientific literature has only scarce data on the enrichment of apple juice with blue honeysuckle berry juice, and hence, it would be interesting to highlight the progress of work in the juice-processing area of the food industry. The growing interest of consumers in fresh beverages with high nutritional value, which are healthy and ready to drink with satisfactory organoleptic properties, encourages producers to introduce new products. This systematic support to develop novel products of consumer and scientific interests has led us to combine two products, one having an established position in the fruit and vegetable market (apple) and the other (raw material) that is currently becoming popular (blue honeysuckle berry). To our knowledge, this is the first report on the physicochemical characteristics of combined apple and berry juices.

The aim of this study was to assess the chemical and sensory properties of mixed apple and blue honeysuckle berry juice. The content of polyphenols in the mixtures of apple juice and blue honeysuckle berry juice was evaluated after a storage period, and a consumer analysis was carried out. In addition, a detailed analysis of the physicochemical parameters (including antioxidant activity, content of anthocyanins, content of vitamin C) was performed.

## 2. Materials and Methods

### 2.1. Reagents and Standards

Acetonitrile, formic acid, phosphoric acid, 2,2′-azino-bis (3-ethylbenzothiazoline-6-sulfonic acid) (ABTS), 6-hydroxy-2,5,7,8-tetramethylchroman-2-carboxylic acid (Trolox), Folin–Ciocalteu reagent, and sodium carbonate were purchased from Sigma-Aldrich (Steinheim, Germany). Cyanidin-3,5-*O*-diglucoside, cyanidin-3-*O*-glucoside, and cyanidin-3-*O*-rutinoside, peonidin-3-*O*-rutinoside and peonidin-3-*O*-glucoside, pelargonidin-3-*O*-glucoside, ascorbic acid, and gallic acid were purchased from Extrasynthese (Lyon, France).

### 2.2. Raw Materials

Blue honeysuckle berries (*L. caerulea* L. cv. Dlinnoplodna) were obtained from the experimental orchard of the Research Institute of Horticulture in Skierniewice, Poland. Apples (*Malus domestica* Borkh. cv. Champion) were obtained from the experimental field of the Department of Pomology of the Warsaw University of Life Sciences, Poland. The blue honeysuckle fruits were harvested in the summer of 2017 and then frozen and stored at –29 °C until the juice was pressed, while the apples were harvested in the autumn of 2017 and then stored at 4 °C until juice extraction.

### 2.3. The Technology of Juice Production

Prior to juice pressing, the raw materials were pretreated. Briefly, the apples were washed and sliced, while the blue honeysuckle berries were thawed at 25 °C. Afterward, the blue honeysuckle berry pulp and apple pulp, at an amount of 0.44 and 1 g/kg, respectively, were enzymatically treated with Rohapect 10 L (AB Enzymes GmbH, Germany). The raw materials thus prepared were pressed using a laboratory press. The obtained apple juice and the blue honeysuckle berry juice were mixed in the proportions of 90:10, 80:20, and 70:30, respectively. Altogether, five different juice variants were produced (Table 1).

The obtained juices were poured into glasses and packed, following which they were pasteurized at 85 °C for 15 min and immediately cooled to 20 °C. The juices were stored at 20 °C, in the absence of light, in order to limit the effect of external factors on the content of the biologically active compounds. All of the obtained juices were analyzed immediately after production and also after 1, 2, 3, and 4 months of storage at 20 °C. The analyses were performed in three replicates.

### 2.4. Analytical Methods

#### 2.4.1. Physicochemical Parameters

The content of total soluble solids (TSS) was measured with the automatic refractometer Refracto 30PX (Mettler Toledo, Poland). For this purpose, a few drops of juice were applied to the prism of the device, and the reading was taken with an accuracy of 0.1. The active acidity (pH) was determined using the pH meter Hi 221 (Hanna Instruments, Poland), which was calibrated using the buffers of pH 4 and 7. The measurement was read from the display of the electronic pH meter with an accuracy of 0.01. To determine the titratable acidity (TTA) of the juice samples, a potentiometric titration with 0.1 M NaOH solution was performed using the pH meter Hi 221 (Hanna Instruments) until a pH of 8.1 was reached. Acidity was expressed in g of malic acid/100 mL of juice, as described by Wojdyło et al. [12]

#### 2.4.2. HPLC Analysis of Anthocyanins

The content of anthocyanins in the tested juices was determined using high-performance liquid chromatography (HPLC) in an isocratic system with a Luna column (5 µm, C18(2), 250 × 4.6 mm, Phenomenex), as described by Goiffon et al. [13]. The flow rate was fixed as 1 mL/min, and the temperature was set at 25 °C. The mobile phase consisted of a mixture of water, acetonitrile, and formic acid at a volume ratio of 810:90:100. Before the analysis, the juices were passed through PTFE syringe filters with a pore size of 0.45 µm. The results were recorded at a wavelength (λ) of 520 nm. The total anthocyanin content was expressed in mg/100 mL of juice.

#### 2.4.3. HPLC Analysis of L-Ascorbic Acid

The content of L-ascorbic acid in the tested juices was determined by applying the HPLC method described by Oszmiański and Wojdyło [14] using an Onyx Monolithic C18 column (100 × 4.6 mm, Phenomenex). The eluent used was 0.1% solution of H_3_PO_4_. Before the analysis, the juices were passed through PTFE syringe filters with a pore size of 0.45 µm. The results were recorded at λ = 254 nm, and the content of L-ascorbic acid was expressed in mg/100 mL of juice.

#### 2.4.4. Analysis of Total Phenolic Content

The determination of total polyphenols (TP) was performed by the method with Folin–Ciocalteu reagent according to Gao et al. [15]. From the calibration curve, the quantities of milligrams of gallic acid corresponding to the investigated absorbance values were calculated. The results are shown in milligrams of gallic acid per 100 mL of juice.

#### 2.4.5. Antioxidant Activity (ABTS^+^) Assay

The antioxidant activity was determined in the juices according to the method of Re et al. [16]. Briefly, 40 µL of juice was taken in tubes. Then, 4 mL of ABTS^+^ cation radical solution was added and the tubes were stirred. Six minutes after the addition of the cation radical solution, the juice samples were taken in 1 cm cuvettes and their absorbance was measured in relation to distilled water at 734 nm using a spectrophotometer (Shimadzu UV—1650PC). The antioxidant activity of the tested extracts was expressed in µmol Trolox/mL [17].

#### 2.4.6. Juice Color Parameters

The color of the juices was analyzed with the Konica Minolta CM-3600d colorimeter equipped with SpectraMagic NX program. Optical cuvettes with an optical path length of 2 mm, 10-degree observer, and D65 illuminant were used for the measurement. The results are presented in the CIE system L*a*b* with an accuracy of 0.01, where the parameter a* describes the share of green or red, parameter b* describes the share of yellow or blue, and the parameter L* corresponds to brightness. On the basis of the measured color parameters (L*, a*, and b*), the total change in color (ΔE) was calculated using the formula Δ = as described by Wojdyło et al. [12].

### 2.5. Sensory Assessment

The sensory evaluation was carried out according to the method described by Lachowicz and Oszmiański [18]. A 5-point hedonic scale was used for evaluation, where 1 indicated fully unacceptable and 5 indicated fully acceptable. The evaluation was carried out by a group of 15 trained panelists. Samples of juices at 20 °C were provided in plastic and transparent cups for evaluation. Parameters such as taste, aroma, color, and general characteristics were assessed.

### 2.6. Statistical Analysis

Statistical analysis of the results was performed using Statistica (version 13.3) and Excel 2016. The standard deviation was calculated using Excel, while Statistica was used to perform analysis of variance (ANOVA) and to analyze the significance of differences using Tukey’s test.

## 3. Results and Discussion

### 3.1. Physicochemical Parameters and Sensory Assessment

TSS value is a quality-control indicator commonly used in the juice industry [18]. The TSS values of juices measured immediately after production ranged from 13.54 (both A and AH1 variants) to 14.15 (H variant) (Table 2). It was found that the storage time had no significant effect on TSS values in any of the variants. The highest TSS values were recorded for the H variant (above 14.05 °Brix). The TSS value seemed to be affected by the degree of fragmentation of fruit tissues during enzymatic treatment. According to Nath et al. [19], a higher degree of tissue breakdown may contribute to a higher TSS value due to a higher release of compounds such as sugars. Compared to apples, blue honeysuckle berries are fleshy and, hence, yield easier to the crushing process.

The TTA and pH are the next important physicochemical parameters that determine the juice quality. They can significantly influence the stability of bioactive components present in fruit juices [11,20]. The results show significant differences in the TTA and pH values between the tested juices. The TTA values measured in juices immediately after production ranged from 0.56 (A juice) to 3.64 g/100 mL (H juice) (Table 2), while the pH values ranged from 2.63 (H juice) to 3.13 (A juice). The addition of blue honeysuckle berry juice in any proportion caused an increase in acidity and a decrease in pH in the apple juice. The higher the proportion of blue honeysuckle berry juice, the higher the TTA value and the lower the pH of the mixed juices (AH1, AH2, AH3).

The TSS/TTA ratio is a parameter that greatly influences the sensory acceptability and consumer preferences. The higher the TSS/TTA ratio of the juice, the sweeter and more acceptable it is by consumers [21]. In this study, it was found that immediately after production, blue honeysuckle berry juice was characterized by an exceptionally low TSS/TTA ratio (3.88), which indicated its lower suitability for direct consumption. By contrast, the apple juice had a higher TSS/TTA ratio (24.28), which justified its high acceptability and popularity among consumers [22]. The addition of the blue honeysuckle berry juice (H variant) to apple juice (A variant) in different proportions caused a decrease in the TSS/TTA ratio. The higher the proportion of H juice added, the lower the TSS/TTA ratio of the mixed juices.

**Figure 2:**
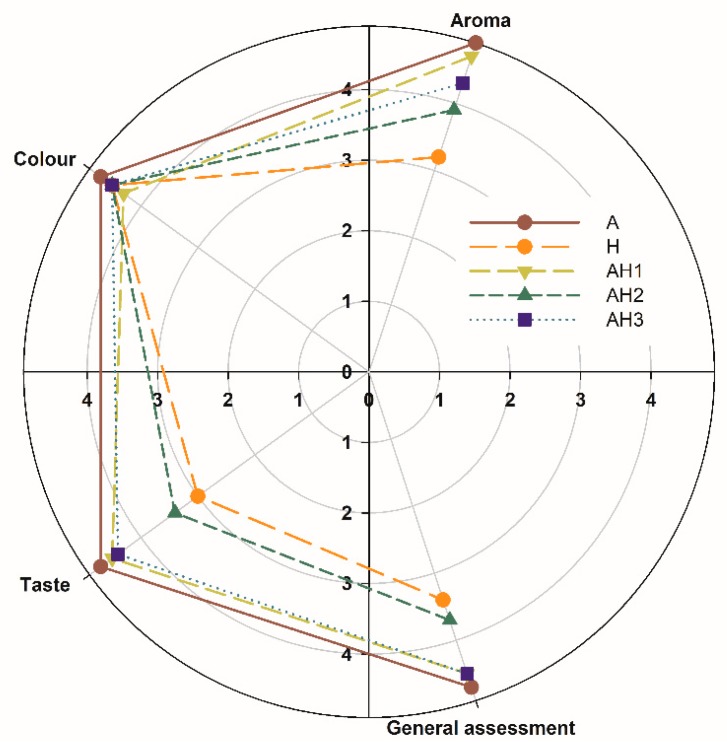
Sensory evaluation of mixed juices; apple and blue honeysuckle berry juice.

To verify the sensory acceptability of the prepared juices, a sensory evaluation was performed using a 5-point hedonic scale (Figure 2). With respect to the aroma, the highest score was given to the A juice (4.9), while the H juice received the lowest score (3.2).

In terms of color, the A juice received the highest score (4.7) and the AH1 juice received the lowest score (4.3). The color of the obtained mixed juices was directly proportional to the percentage of H juice added. Visually, the color of the AH1 juice was the most light compared to the other mixed juices and the most different from the base 100% blue honeysuckle berry juice (H), which is also indicated by the results of parameter measurements colors using the CieLab method, where the AH1 juice showed the highest value of the L* parameter compared to other mixed juices and H juice. With respect to taste, the A juice achieved the highest score (4.7), which was probably due to the lowest acidity value, higher pH, highest TSS/TTA ratio, and the fact that it is the commonly known fruit juice and widely accepted by consumers [22], whereas the H juice received the lowest score (3.0), which was probably due to its very high acidity. Both the AH1 and AH3 juice variants received relatively high scores in terms of taste (4.5 and 4.4, respectively), which indicates that the mixed juices with 10% and 30% proportions of the blue honeysuckle berry juice would be acceptable by consumers. However, as regards taste, the variant with 20% of blue honeysuckle berry juice (AH2) received the lowest score (3.4), which confirms the fact that the percentages of individual juices should be optimally selected for the production of mixed juices. According to Lesschaeve and Noble [23], the sensory characteristics of beverages can be influenced by factors such as polyphenolic composition, pH, and sugar content. However, despite a lower pH and higher TSS/TTA ratio compared to the AH3 juice, the AH2 juice was less sensorially acceptable, mainly in terms of taste and aroma. In this study, fruit juices with different chemical compositions and polyphenol contents [8,22] were mixed, which could have led to interactions between individual components influencing the sensory features.

Polysaccharides may reduce the perception of tart taste. Astringent properties of plant extracts rich in polyphenols reduced the use of polydextrose [24] and carboxymethylcellulose [25]. The reduction of the astringent effect may be the result of the adsorption of polyphenols on the surface of polysaccharides [25]. Apples are known for their polysaccharide content, especially from the pectic polysaccharides group. It has been shown that polysaccharides contained in apple tissue can interact with polyphenols through hydrophobic interactions and hydrogen bonds [26]. Mixing blue honeysuckle berry juice containing a high content of polyphenols (767.88 mg/100 mL) with apple juice with a high content of polysaccharides could lead to mutual interactions between polysaccharides and polyphenolic compounds, and thus give an effect that reduces the astringency, bitterness, and acidity of H juice. This means that contrary to the earlier quoted studies [24,25], the reduction of unfavorable astringency and bitterness can be achieved not only by adding specific polysaccharides, but also by mixing juices from raw materials differing in the content of these ingredients.

A number of compounds have been identified in the blue honeysuckle berry fruit that can affect the perception of the taste of this raw material. In the study of Wojdyło et al. [27], it was shown that blue honeysuckle berry fruit contains a high content of proanthocyanidins, and therefore, compounds belonging to the tannin group. Proanthocyanidins are compounds with proven effects on sensory characteristics of food. It has been shown that above all, they are compounds responsible for astringency, and they can also shape bitterness and sourness of plant raw materials [28]. In addition, recent studies have shown that iridoids (compounds from the monoterpenes group) are present in the blue honeysuckle berry fruit, which, as plant defense substances, give bitterness to plant raw materials [10,29,30]. In addition, chlorogenic acids are the dominant group of phenolic acids in blue honeysuckle berry fruits, and it has been shown that these secondary metabolites shape the astringency and bitterness of a coffee beverage [26,31].

Thus, this study disproved the notion that the higher the percentage of the more acidic juice and the lower the TSS/TTA ratio, the lesser the sensory acceptability. Therefore, further research should be aimed at understanding the effect of the percentage shares of individual juices in mixed juices on sensory characteristics and the interactions between the components of juices. This is important to improve the health benefits and sensory characteristics in the design of functional foods [32,33,34,35].

### 3.2. L-Ascorbic Acid Content

Vitamin C is a well-known and essential vitamin in the human diet. It acts as a natural antioxidant, preventing oxidative stress in the body [36]. Vitamin C consists of L-ascorbic acid and dehydroascorbic acid, an oxidized form of ascorbic acid [37]. In this study, the content of L-ascorbic acid in all of the juice variants was determined, and significant differences were observed (Table 2). Immediately after production, the highest content of L-ascorbic acid was observed in the H juice and the lowest in the A juice (32.59 and 0.52 mg/100 mL, respectively). In the case of the mixed juices (AH1, AH2, AH3), the higher the content of H juice, the higher the content of L-ascorbic acid found. However, after 4 months of storage, a high degree of acid degradation was observed. The lowest loss was found in AH3 juice, in which 27% of the initial content of L-ascorbic acid remained, and the highest loss was found in AH1 juice, in which only 18% of the initial content remained. The factors that caused the degradation of L-ascorbic acid observed in this study may be oxygen residue and high temperature maintained during juice pasteurization [38,39]. Vitamin C is a component with functional properties, but unfortunately, it is very sensitive to the thermal treatment applied during processing [39,40].

### 3.3. Anthocyanin Content

Anthocyanins are natural plant pigments that belong to the family of polyphenolic compounds classified under flavonoids. Plant raw materials containing anthocyanins are often red, pink, blue, or black in color [41,42]. Anthocyanins are also bioactive compounds that are beneficial to humans [31]. An advantage of the blue honeysuckle berry is a very high content of anthocyanins, ranging from 400 to 1500 mg/100 g [43]. Many studies have indicated that anthocyanins are health-promoting compounds, due to their antioxidant, anticancer, neuroprotective, and cardiovascular-supporting properties, among others [42,44,45,46,47]. In contrast, apples have a lesser amount of anthocyanins, and depending on the variety, the content of these compounds may vary from 0 to around 27 mg/100 g [22,23]. In our study, anthocyanins were not identified in juice A (Table 3).

This may have been due to the fact that anthocyanins are present in apple skin, which is removed to a large extent as a by-product after the pressing process, and thus, even though these compounds are present in the fruits, they might not be transferred to the juice. Therefore, the addition of blue honeysuckle berry juice may be an excellent option to enrich the apple juice with prohealth anthocyanins. The content of anthocyanins in the H juice measured immediately after production was, on average, 595.39 mg/100 mL. The higher the addition of the blue honeysuckle berry juice, the higher the average anthocyanin content detected in the mixed juices. Immediately after mixing, the highest content of anthocyanins was recorded in the AH3 juice (186.37 mg/100 mL) and the lowest in the AH1 juice (49.86 mg/100 mL) among the mixed juices. During the 4 months of storage, the total anthocyanin content was found to be reduced in the juices. The AH3 juice contained only 45% of the initial anthocyanin content, while the AH1 juice contained only 34%. However, in the H juice, 64% of the initial anthocyanin content remained. Thus, the higher the share of blue honeysuckle berry juice, the higher the total anthocyanin content and the lower the losses during storage. Anthocyanins are compounds, the stability of which is determined by the pH value [11].

A higher level of retention of total anthocyanins in the AH3 juice may be due to the fact that its pH value (below 3.0) was favorable for anthocyanins’ stability during the whole storage period. In our study, six types of anthocyanins were identified: Cyanidin-3,5-*O*-diglucoside, cyanidin-3-*O*-glucoside, and cyanidin-3-*O*-rutinoside, peonidin-3-*O*-rutinoside and peonidin-3-*O*-glucoside, and pelargonidin-3-*O*-glucoside. Among these anthocyanins, the most abundant in the analyzed juices was cyanidin-3-*O*-glucoside. This is in accordance with other studies which showed that cyanidin-3-*O*-glucoside is the anthocyanin found in the highest amount in the blue honeysuckle berry [11]. Its content in the AH1 and AH3 juices was estimated to be 34.33 and 143.96 mg/100 mL, respectively. During the storage, the content of cyanidin-3-*O*-glucoside decreased, and after 4 months of storage, there remained only 48% and 36% of the initial content in AH3 and AH1 juices, respectively. This phenomenon could be explained by copigmentation. The effectiveness of a copigmentation reaction in reducing anthocyanin degradation depends on the type and concentration of compounds involved in the reaction. Depending on the dye and copigment structure, pH, temperature, and storage time, the course of copigmentation changes, which leads to an increase in absorbance and increases the color intensity [48].

### 3.4. Total Polyphenols and Antioxidant Activity

The polyphenol content of juice depends mostly on the raw material used. In the present study, it was found that the content of TP differed significantly in the juice variants. One of the aims of this study was to enrich the apple juice, which is poorer in polyphenols (A juice—47.39 mg/100 mL) with blue honeysuckle berry juice, which is rich in polyphenols (H juice—767.88 mg/100 mL).

Enrichment with H juice increased the TP value of A juice (by 2.7, 3.5, and 6.1 times in AH1, AH2, and AH3, respectively). It was observed that the storage time also had a significant effect on the TP value of all the juices tested (Table 4).

After 4 months of storage, the content of polyphenols was 28.65 and 635.80 mg/100 mL in the A and H juices, respectively. Among the mixed juices, the AH3 juice was characterized by the highest content of TP after 4 months of storage (174.60 mg/100 mL). Additionally, the highest losses of TP content were recorded in the AH3 juice (only 61% of the initial content remained), whereas 67% and 76% of TP content remained in the AH1 and AH2 juices, respectively. Nevertheless, despite the highest losses during the 4 months of storage, the AH3 juice had 2.0 and 1.4 times higher TP content in comparison to the AH1 and AH2 juices, respectively. Enrichment by blue honeysuckle berry juice also contributed to the increase of antioxidant activity in apple juice.

An increase in the free radical-capturing capacity by 5.3, 6.2, and 7.6 times was observed in AH1, AH2, and AH3 juices, respectively, after production in comparison to the A juice. Immediately after production, the antioxidant activity was determined to be 20.79 and 30.09 µmol Trolox/mL in the AH1 and AH3 juices, respectively. During storage, the antioxidant activity decreased similar to the content of TP. The research of Lachowicz and Oszmiański [18] also proved that the addition of cranberrybush juice to pear juice resulted in a significant increase in its TP content and an improvement in antioxidant properties. This confirms that the use of fruit juices which are sensorially less acceptable, but having a high antioxidant capacity, even in small amounts, in the production of mixed juices based on juices from popular fruits (e.g., apples or pears) enables obtaining a product more abundant in polyphenols with potential functional properties.

### 3.5. Juice Color Parameters

Color is an essential quality characteristic that influences the acceptability of fruit juices [12]. It was found that the color parameters L*, a*, and b* significantly differed among the studied juices (Table 5). Immediately after production, the value of L* parameter, indicating the brightness level, was measured as 4.47 and 98.28 in the H and A juice, respectively. In the case of mixed juices (AH1, AH2, AH3), the higher the addition of H juice, the lower the value of the L* parameter and thus the lower the brightness of the tested juices. In addition, a significant effect of the storage time on the value of L* parameter was found in all the juices except the A variant. As the storage time passed, an increase in brightness was observed in the AH1, AH2, AH3, and H juices.

Immediately after production, the value of the a* parameter was measured as 0.21 and 65.79 in the A and AH3 juices, respectively. During 4 months of storage, there was a decrease in the value of this parameter in all the tested juices, with the exception of the juice A. The decrease in the share of red color resulted from the degradation of anthocyanins, which determine the color of berry juices [49]. Immediately after production, the values of the parameter b* were measured as 1.63 and 39.22 in the juices A and AH3, respectively. Moreover, during storage, the share of yellow color increased in all the studied juices. After 4 months of storage, the juices analyzed were brighter and more yellow and less red in color than the juices analyzed immediately after production. The parameter ΔE indicated the change in color during storage and expressed the possibility of distinguishing the difference in color by the human eyesight. It is considered that when ΔE value is less than 1, the color difference is not perceived; when ΔE is between 1 and 2, the color change is noticed by an experienced observer; when ΔE is between 2 and 3.5, the color change might be perceived by the consumer; when ΔE is between 3.5 and 5, the consumer can observe a clear color difference between the products; and when ΔE is above 5, the consumer has the impression of a completely distinct color [18]. After 1 month of storage, the ΔE values of the juices A and H reached 0.43 and 0.5, respectively, which meant that there was no change in color in these juice variants. On the other hand, in the case of mixed juices, the value of ΔE was 0.55 and 3.09 in the AH3 and AH2 juices, respectively. After 4 months of storage, the ΔE value of only the A juice did not exceed 2 (1.91), whereas in the other tested juices, the ΔE value exceeded 5, which indicates that after 4 months of storage, the color of the juice differed significantly from the color exhibited by the juices immediately after production. This is in line with the findings reported by other researchers that storage is one of the main determinants affecting the color of juices [50,51]. For instance, in the study of Lachowicz and Oszmiański [18], it was found that after 5 months of storage at 25 °C, an increase in ΔE was found in the pear juices mixed with cranberrybush juice.

## 4. Conclusions

The results of this study indicate that the addition of blue honeysuckle berry juice can enrich the apple juice with anthocyanins, and thus improve its prohealth properties. Mixing with apple juice is also a very good way to utilize blue honeysuckle berry juice that is otherwise unpreferred by consumers due to its very high level of acidity. An advantage of the blue honeysuckle juice is undoubtedly its higher content of anthocyanins than in juices obtained from other popular fruits. This makes blue honeysuckle berry particularly valuable in the production of functional juices. To the best of our knowledge, this work is the first to deal with the influence of different doses of blue honeysuckle berry juice on the quality characteristics of mixed apple–blue honeysuckle berry juices. Therefore, this study is crucial in the current pursuit of new raw materials for the production of functional foods and in the future design of similar products on an industrial scale.

## Figures and Tables

**Table 1:** The types of juices obtained.

Symbol of the Juice Type	The Percentage Share of the Individual Juice [%]
A	100% A
H	100% H
AH1	90% A-10% H
AH2	80% A-20% H
AH3	70% A-30% H

A Juice from apple; H Juice from blue honeysuckle berry.

**Table 2:** Physicochemical parameters and L-ascorbic acid content in the tested juices after production and during 4 months of storage.

Juice	Parameters	Time of Storage				
		After Production	1 Month	2 Months	3 Months	4 Months
A	TSS ^A^	13.54 ± 0.08 ^a^	13.44 ± 0.00 ^a^	13.50 ± 0.00 ^a^	13.44 ±0.08 ^a^	13.54 ± 0.08 ^a^
	TTA ^B^	0.56 ± 0.00 ^a^	0.54 ± 0.00 ^b^	0.50 ± 0.00 ^c^	0.49 ± 0.00 ^c^	0.50 ± 0.00 ^c^
	TSS/TTA	24.17	24.88	27.00	27.42	27.08
	pH	3.13 ± 0.01 ^c^	3.19 ± 0.01 ^b^	3.24 ± 0.02 ^b^	3.19 ± 0.02 ^b^	3.51 ± 0.02 ^a^
	L-ascorbic acid ^C^	0.52 ± 0.00 ^a^	0.44 ± 0.00 ^b^	0.31 ± 0.00 ^c^	0.24 ± 0.00 ^d^	0.17 ± 0.01 ^e^
H	TSS ^A^	14.15 ± 0.08 ^a,b^	14.05 ± 0.08 ^b^	14.25 ± 0.08 ^a^	14.15 ±0.08 ^a,b^	14.12 ± 0.02 ^a,b^
	TTA ^B^	3.64 ± 0.02 ^a^	3.63 ± 0.02 ^a^	3.59 ± 0.02 ^a,b^	3.56 ± 0.02 ^b^	3.56 ± 0.02 ^b^
	TSS/TTA	3.88	3.87	3.96	3.97	3.96
	pH	2.63 ± 0.00 ^c^	2.67 ± 0.00 ^b^	2.68 ± 0.00 ^b^	2.68 ± 0.01 ^b^	2.72 ± 0.00 ^a^
	L-ascorbic acid ^C^	32.59 ± 0.19 ^a^	11.98 ± 0.07 ^b^	11.62 ± 0.07 ^c^	10.44 ± 0.06 ^d^	9.36 ± 0.05 ^e^
AH1	TSS ^A^	13.54 ± 0.08 ^a^	13.53 ± 0.08 ^a^	13.54 ± 0.08 ^a^	13.54 ± 0.08 ^a^	13.64 ± 0.08 ^a^
	TTA	0.78 ± 0.01 ^a^	0.76 ± 0.00 ^b^	0.74 ± 0.01 ^c^	0.76 ± 0.00 ^b^	0.69 ± 0.00 ^d^
	TSS/TTA	17.35	17.80	18.29	17.81	19.76
	pH	3.02 ± 0.01 ^c^	3.08 ± 0.00 ^b^	3.08 ± 0.00 ^b^	3.08 ± 0.02 ^b^	3.40 ± 0.01 ^a^
	L-ascorbic acid ^C^	1.14 ± 0.01 ^a^	0.86 ± 0.00 ^b^	0.52 ± 0.00 ^c^	0.40 ± 0.00 ^d^	0.20 ± 0.04 ^e^
AH2	TSS ^A^	13.64 ± 0.08 ^a^	13.58 ± 0.00 ^a^	13.54 ± 0.08 ^a^	13.64 ± 0.08 ^a^	13.64 ± 0.08 ^a^
	TTA ^B^	1.08 ± 0.01 ^a^	1.06 ± 0.01 ^b^	1.03 ± 0.01 ^c^	1.08 ± 0.01 ^a^	0.97 ± 0.01 ^d^
	TSS/TTA	12.62	12.81	13.14	12.62	14.06
	pH	2.91 ± 0.01 ^d^	2.97 ± 0.02 ^c^	2.98 ± 0.00 ^c^	3.19 ± 0.02 ^b^	3.31 ± 0.01 ^a^
	L-ascorbic acid ^C^	4.23 ± 0.02 ^a^	2.58 ± 0.01 ^b^	1.45 ± 0.01 ^c^	1.14 ± 0.01 ^d^	0.96 ± 0.05 ^e^
AH3	TSS ^A^	13.81 ± 0.08 ^a^	13.95 ± 0.08 ^a^	13.95 ± 0.07 ^a^	13.75 ± 0.08 ^a^	13.95 ± 0.08 ^a^
	TTA ^B^	1.34 ± 0.01 ^a^	1.33 ± 0.01 ^a^	1.21 ± 0.01 ^d^	1.27 ± 0.01 ^b^	1.24 ± 0.01 ^c^
	TSS/TTA	10.30	10.48	11.52	10.82	11.25
	pH	2.86 ± 0.01 ^c^	2.90 ± 0.00 ^b c^	2.94 ± 0.01 ^b^	2.90 ± 0.01 ^b c^	3.25 ± 0.01 ^a^
	L-ascorbic acid ^C^	8.07 ± 0.05 ^a^	6.10 ± 0.04 ^b^	3.47 ± 0.02 ^c^	2.89 ± 0.57 ^c^	2.14 ± 0.01 ^d^

^A^ TSS total soluble solids [°Brix]; ^B^ TTA total titratable acidity [g of malic acid/100 mL]; ^C^ L-ascorbic acid [mg/100 ml]; ^a–e^ Means with the same letter did not differ significantly.

**Table 3:** Content of anthocyanins [mg/100 mL] in the tested juices immediately after production and during 4 months of storage.

Juice	Parameters	Time of Storage				
		After Production	1 Month	2 Months	3 Months	4 Months
A	Total anthocyanins	0.0 ± 0.0 ^a^	0.0 ± 0.0 ^a^	0.0 ± 0.0 ^a^	0.0 ± 0.0 ^a^	0.0 ± 0.0 ^a^
H	Cyanidin 3,5-*O*-diglucoside	60.27 ± 0.47 ^a^	47.93 ± 0.51 ^b^	45.75 ± 0.25 ^c^	35.02 ± 1.44 ^d^	30.88 ± 0.15 ^e^
	Cyanidin 3-*O*-glucoside	460.26 ± 2.60 ^a^	359.38 ± 2.98 ^b^	340.18 ± 3.40 ^c^	337.68 ± 12.63 ^c^	305.54 ± 1.62 ^d^
	Cyanidin 3-*O*-rutinoside	35.19 ± 0.31 ^a^	29.34 ± 0.35 ^b^	26.36 ± 0.36 ^c^	25.43 ± 1.09 ^c^	23.06 ± 0.15 ^d^
	Pelargonidin 3-*O*-glucoside	9.32 ± 0.13 ^a^	9.14 ± 0.06 ^a,b^	8.80 ± 0.06 ^b c^	8.40 ± 0.38 ^c^	5.30 ± 0.03 ^d^
	Peonidin 3-*O*-glucoside	23.71 ± 0.22 ^a^	20.29 ± 0.22 ^b^	18.70 ± 0.49 ^c^	17.28 ± 0.72 ^d^	15.38 ± 0.10 ^e^
	Peonidin 3-*O*-rutinoside	6.63 ± 0.06 ^a^	5.31 ± 0.33 ^b^	4.29 ± 0.11 ^c^	3.51 ± 0.04 ^d^	3.38 ± 0.05 ^d^
	Total anthocyanins	595.39 ± 3.77 ^a^	471.39 ± 4.44 ^b^	444.09 ± 3.74 ^c^	427.32 ± 5.28 ^d^	383.56 ± 2.09 ^e^
AH1	Cyanidin 3,5-*O*-diglucoside	6.48 ± 0.10 ^a^	5.07 ± 0.03 ^b^	3.38 ± 0.05 ^c^	2.95 ± 0.08 ^d^	2.62 ± 0.02 ^e^
	Cyanidin 3-*O*-glucoside	34.33 ± 0.84 ^a^	17.12 ± 0.61 ^b^	15.90 ± 0.16 ^b^	14.15 ± 0.89 ^c^	12.49 ± 0.17 ^c^
	Cyanidin 3-*O*-rutinoside	4.56 ± 0.36 ^a^	2.16 ± 0.01 ^b^	1.15 ± 0.02 ^c^	0.95 ± 0.02 ^c^	0.81 ± 0.01 ^c^
	Pelargonidin 3-*O*-glucoside	1.13 ± 0.02 ^a^	1.15 ± 0.01 ^a^	1.09 ± 0.01 ^b^	0.96 ± 0.01 ^c^	0.58 ± 0.01 ^d^
	Peonidin 3-*O*-glucoside	3.17 ± 0.07 ^a^	1.34 ± 0.04 ^b^	1.29 ± 0.01 ^b^	0.62 ± 0.01 ^c^	0.34 ± 0.01 ^d^
	Peonidin 3-*O*-rutinoside	0.18 ± 0.01 ^a^	0.16 ± 0.00 ^b^	0.15 ± 0.00 ^b c^	0.15 ± 0.01 ^c^	0.10 ± 0.00 ^d^
	Total anthocyanins	49.86 ± 1.36 ^a^	27.00 ± 0.72 ^b^	22.97 ± 1.92 ^c^	19.77 ± 1.03 ^d^	16.94 ± 0.33 ^e^
AH2	Cyanidin 3,5-*O*-diglucoside	9.37 ± 0.08 ^b^	10.10 ± 0.10 ^a^	5.95 ± 0.04 ^c^	5.90 ± 0.05 ^c^	5.59 ± 0.10 ^d^
	Cyanidin 3-*O*-glucoside	68.76 ± 0.41 ^a^	31.83 ± 0.16 ^b^	30.63 ± 0.31 ^c^	27.62 ± 0.18 ^d^	25.55 ± 0.13 ^e^
	Cyanidin 3-*O*-rutinoside	5.04 ± 0.03 ^a^	4.82 ± 0.03 ^b^	2.86 ± 0.02 ^c^	2.65 ± 0.02 ^d^	2.62 ± 0.01 ^d^
	Pelargonidin 3-*O*-glucoside	1.20 ± 0.01 ^a^	1.30 ± 0.07 ^a^	0.75 ± 0.01 ^b^	0.79 ± 0.05 ^b^	0.80 ± 0.05 ^b^
	Peonidin 3-*O*-glucoside	3.28 ± 0.02 ^a^	2.85 ± 0.03 ^b^	1.55 ± 0.01 ^c^	1.41 ± 0.07 ^d^	1.29 ± 0.07 ^e^
	Peonidin 3-*O*-rutinoside	0.58 ± 0.00 ^a^	0.54 ± 0.00 ^b^	0.39 ± 0.00 ^c^	0.36 ± 0.00 ^d^	0.27 ± 0.00 ^e^
	Total anthocyanins	88.22 ± 0.52 ^a^	51.45 ± 0.38 ^b^	42.12 ± 0.22 ^c^	38.73 ± 0.34 ^d^	36.11 ± 0.35 ^e^
AH3	Cyanidin 3,5-*O*-diglucoside	20.17 ± 0.19 ^a^	18.59 ± 0.11 ^b^	12.31 ± 0.06 ^c^	11.55 ± 0.07 ^d^	6.74 ± 0.08 ^e^
	Cyanidin 3-*O*-glucoside	143.96 ± 0.96 ^a^	91.77 ± 0.58 ^b^	87.87 ± 0.87 ^c^	84.79 ± 0.51 ^c^	68.89 ± 2.09 ^d^
	Cyanidin 3-*O*-rutinoside	11.12 ± 0.06 ^a^	10.76 ± 0.07 ^b^	7.65 ± 0.05 ^c^	7.38 ± 0.15 ^d^	4.43 ± 0.08 ^e^
	Pelargonidin 3-*O*-glucoside	2.71 ± 0.03 ^a^	2.76 ± 0.11 ^a^	2.03 ± 0.06 ^b^	1.78 ± 0.10 ^c^	1.15 ± 0.01 ^d^
	Peonidin 3-*O*-glucoside	7.51 ± 0.04 ^a^	6.73 ± 0.09 ^a^	4.51 ± 0.09 ^b^	3.94 ± 0.16 ^b^	2.78 ± 0.82 ^c^
	Peonidin 3-*O*-rutinoside	0.90 ± 0.03 ^a^	0.88 ± 0.01 ^a^	0.50 ± 0.06 ^b^	0.43 ± 0.01 ^b c^	0.39 ± 0.00 ^c^
	Total anthocyanins	186.37 ± 1.3 ^a^	131.49 ± 0.95 ^b^	114.87 ± 1.11 ^c^	109.87 ± 0.99 ^d^	84.38 ± 3.08 ^e^

^a–e^ Means with the same letter did not differ significantly.

**Table 4:** Total polyphenols (TP) [mg/100 mL] and antioxidant activity (ABTS) [μmol Trolox/mL] in juices immediately after production and during 4 months of storage.

Juice	Parameters	Time of Storage				
		After Production	1 Month	2 Months	3 Months	4 Months
A	TP	47.39 ± 0.47 ^a^	42.80 ± 0.43 ^b^	41.28 ± 0.41 ^c^	32.21 ± 0.32 ^d^	28.65 ± 0.29 ^e^
	ABTS	3.94 ± 0.00 ^a^	3.54 ± 0.02 ^b^	3.17 ± 0.03 ^c^	2.61 ± 0.18 ^d^	2.26 ± 0.04 ^e^
H	TP	767.88 ± 7.68 ^a^	762.80 ± 7.63 ^a^	750.10 ± 7.50 ^a^	706.92 ± 7.07 ^b^	635.80 ± 6.36 ^c^
	ABTS	96.44 ± 0.48 ^a^	95.24 ± 0.47 ^a,b^	92.68 ± 1.11 ^b^	88.15 ± 1.87 ^c^	61.90 ± 0.57 ^d^
AH1	TP	128.07 ± 0.76 ^a^	115.98 ± 1.16 ^b^	108.98 ± 1.09 ^c^	94.07 ± 0.94 ^d^	85.61 ± 0.86 ^e^
	ABTS	20.79 ± 0.56 ^a^	17.31 ± 0.23 ^b^	10.87 ± 0.88 ^c^	10.22 ± 0.12 ^c,d^	8.95 ± 0.19 ^d^
AH2	TP	165.50 ± 4.47 ^a^	143.77 ± 1.44 ^b^	135.41 ± 1.35 ^c^	129.45 ± 1.29 ^c,d^	126.00 ± 1.26 ^d^
	ABTS	24.8 ± 0.76 ^a^	19.25 ± 1.24 ^b^	15.41 ± 0.50 ^c^	13.75 ± 0.62 ^c^	10.68 ± 0.08 ^d^
AH3	TP	287.54 ± 2.37 ^a^	258.71 ± 1.98 ^b^	193.70 ± 1.93 ^c^	186.07 ± 1.86 ^d^	174.60 ± 1.76 ^e^
	ABTS	30.09 ± 0.15 ^a^	27.52 ± 0.31 ^b^	26.46 ± 0.13 ^c^	24.49 ± 0.30 ^d^	22.09 ± 0.33 ^e^

^a–e^ Means with the same letter did not differ significantly.

**Table 5:** Colour parameters of the tested juices immediately after production and during 4 months of storage.

Juice	Parameters	Time of Storage				
		After Production	1 Month	2 Months	3 Months	4 Months
A	L*	98.28 ± 0.98 ^a^	98.28 ± 0.98 ^a^	98.35 ± 0.98 ^a^	98.61 ± 0.99 ^a^	98.87 ± 0.99 ^a^
	a*	0.21 ± 0.00 ^a^	0.17 ± 0.00 ^b^	0.21 ± 0.00 ^a^	0.17 ± 0.00 ^b^	0.15 ± 0.00 ^c^
	b*	1.63 ± 0.02 ^d^	2.06 ± 0.02 ^c^	2.86 ± 0.03 ^b^	3.52 ± 0.04 ^a^	3.45 ± 0.03 ^a^
	ΔE	-	0.43	1.23	1.92	1.91
H	L*	4.47 ± 0.04 ^d^	4.71 ± 0.05 ^c^	5.44 ± 0.05 ^b^	6.30 ± 0.06 ^a^	6.40 ± 0.06 ^a^
	a*	35.34 ± 0.35 ^a^	35.11 ± 0.35 ^a^	32.97 ± 0.33 ^b^	30.29 ± 0.30 ^c^	29.22 ± 0.29 ^d^
	b*	7.66 ± 0.08 ^d^	8.03 ± 0.08 ^c^	9.35 ± 0.09 ^b^	10.81 ± 0.11 ^a^	10.91 ± 0.11 ^a^
	ΔE	-	0.50	3.07	6.23	7.19
AH1	L*	49.59 ± 0.50 ^c^	50.76 ± 0.51 ^b c^	51.81 ± 0.52 ^b^	51.96 ± 0.52 ^b^	53.48 ± 0.53 ^a^
	a*	57.98 ± 0.70 ^a^	58.28 ± 0.58 ^a^	56.95 ± 0.57 ^a^	57.29 ± 0.57 ^a^	55.16 ± 0.55 ^b^
	b*	10.04 ± 0.10 ^e^	11.78 ± 0.12 ^d^	14.48 ± 0.14 ^c^	20.22 ± 0.20 ^b^	33.77 ± 0.34 ^a^
	ΔE	-	2.12	5.07	10.48	24.21
AH2	L*	33.35 ± 0.33 ^d^	34.83 ± 0.35 ^c^	34.84 ± 0.35 ^c^	36.39 ± 0.36 ^b^	38.55 ± 0.39 ^a^
	a*	60.05 ± 3.44 ^a^	60.38 ± 0.60 ^a^	55.27 ± 0.55 ^b^	52.97 ± 0.53 ^b c^	49.59 ± 0.50 ^c^
	b*	29.64 ± 0.30 ^e^	32.33 ± 0.32 ^d^	37.92 ± 0.38 ^c^	46.04 ± 0.46 ^b^	59.34 ± 0.59 ^a^
	ΔE	-	3.09	9.68	18.12	31.92
AH3	L*	24.10 ± 0.24 ^c^	24.29 ± 0.38 ^c^	25.39 ± 0.25 ^b^	26.39 ± 0.26 ^a^	27.26 ± 0.27 ^a^
	a*	65.79 ± 0.66 ^a^	65.28 ± 1.77 ^a^	61.57 ± 0.62 ^b^	62.30 ± 0.70 ^b^	60.95 ± 0.71 ^b^
	b*	39.22 ± 0.39 ^d^	39.31 ± 0.42 ^d^	42.69 ± 0.42 ^c^	44.91 ± 0.44 ^b^	46.88 ± 0.46 ^a^
	ΔE	-	0.55	5.61	7.06	9.60

^a–e^ Means with the same letter did not differ significantly.

## References

[1] Snyder F., Ni L. (2017). Chinese apples and the emerging world food trade order: Food safety, international trade, and regulatory collaboration between China and the European Union. Chin. J. Comp. Law (CJCL).

[2] Oliveira B.G., Tosato F., Folli G.S., de Leite J.A., Ventura J.A., Endringer D.C., Filgueiras P.R., Romão W. (2019). Controlling the quality of grape juice adulterated by apple juice using ESI (-) FT-ICR mass spectrometry. Microchem. J..

[3] Persic M., Mikulic-Petkovsek M., Slatnar A., Veberic R. (2017). Chemical composition of apple fruit, juice and pomace and the correlation between phenolic content, enzymatic activity and browning. LWT Food Sci. Technol..

[4] Barreira J.C., Arraibi A.A., Ferreira I.C. (2019). Bioactive and functional compounds in apple pomace from juice and cider manufacturing: Potential use in dermal formulations. Trends Food Sci. Technol..

[5] Senica M., Stampar F., Veberic R., Mikulic-Petkovsek M. (2019). Cyanogenic glycosides and phenolics in apple seeds and their changes during long term storage. Sci. Hortic..

[6] Molina A.K., Vega E.N., Pereira C., Dias M.I., Heleno S.A., Rodrigues P., Fernandes I.F., Barreiro M.F., Kostić M., Soković M. (2019). Promising antioxidant and antimicrobial food colourants from *Lonicera caerulea* L. var. *Kamtschatica*. Antioxidants.

[7] Auzanneau N., Weber P., Kosińska-Cagnazzo A., Andlauer W. (2018). Bioactive compounds and antioxidant capacity of *Lonicera caerulea* berries: Comparison of seven cultivars over three harvesting years. J. Food Compos. Anal..

[8] Becker R., Szakiel A. (2019). Phytochemical characteristics and potential therapeutic properties of blue honeysuckle *Lonicera caerulea* L. (*Caprifoliaceae*). J. Herb. Med..

[9] Senica M., Stampar F., Mikulic-Petkovsek M. (2019). Blue honeysuckle (*Lonicera cearulea* L. subs. *edulis*) berry; A rich source of some nutrients and their differences among four different cultivars. Sci. Hortic..

[10] Oszmiański J., Kucharska A.Z. (2018). Effect of pre-treatment of blue honeysuckle berries on bioactive iridoid content. Food Chem..

[11] Rupasinghe H.V., Arumuggam N., Amararathna M., De Silva A.B.K.H. (2018). The potential health benefits of haskap (*Lonicera caerulea* L.): Role of cyanidin-3-O-glucoside. J. Funct. Foods.

[12] Wojdyło A., Teleszko M., Oszmiański J. (2014). Physicochemical characterisation of quince fruits for industrial use: Yield, turbidity, viscosity and colour properties of juices. Int. J. Food Sci. Technol..

[13] Goiffon J.-P., Mouly P.P., Gaydou E.M. (1999). Anthocyanic pigment determination in red fruit juices, concentrated juices and syrups using liquid chromatography. Anal. Chim. Acta.

[14] Oszmiański J., Wojdyło A. (2009). Effects of blackcurrant and apple mash blending on the phenolics contents, antioxidant capacity, and colour of juices. Czech. J. Food Sci..

[15] Gao X., Ohlander M., Jeppsson N., Bjork I., Trajkowski V. (2000). Changes in antioxidant effects and their relationship to phytonutrients in fruits of sea buckthorn (*Hippophae rhamnoides* L.) during maturation. J. Agric. Food Chem..

[16] Re R., Pellegrini N., Proteggente A., Pannala A., Yang M., Rice-Evans C. (1999). Antioxidant activity applying an improved ABTS radical cation decolorization assay. Free Radic. Biol. Med..

[17] Cendrowski A., Ścibisz I., Mitek M., Kieliszek M. (2018). Influence of harvest seasons on the chemical composition and antioxidant activity in *Rosa rugosa* petals. Agrochimica.

[18] Lachowicz S., Oszmiański J. (2018). The influence of addition of cranberrybush juice to pear juice on chemical composition and antioxidant properties. J. Food Sci. Technol..

[19] Nath P., Varghese E., Kaur C. (2015). Optimization of enzymatic maceration for extraction of carotenoids and total phenolics from sweet pepper using response surface methodology. Indian J. Hortic..

[20] Islam M., Ahmad I., Ahmed S., Sarker A. (2014). Biochemical Composition and shelf life study of mixed fruit juice from orange & pineapple. J. Environ. Sci. Nat. Resour..

[21] Jaros D., Thamke I., Raddatz H., Rohm H. (2009). Single-cultivar cloudy juice made from table apples: An attempt to identify the driving force for sensory preference. Eur. Food Res. Technol..

[22] Francini A., Sebastiani L. (2013). Phenolic compounds in apple (*Malus* x *domestica* Borkh.): Compounds characterization and stability during postharvest and after processing. Antioxidants.

[23] Lesschaeve I., Noble A.C. (2005). Polyphenols: Factors influencing their sensory properties and their effects on food and beverage preferences. Am. J. Clin. Nutr..

[24] Sunarharum W.B., Williams D.J., Smyth H.E. (2014). Complexity of coffee flavor: A compositional and sensory perspective. Food Res. Int..

[25] Troszyńska A., Narolewska O., Robredo S., Estrella I., Hernández T., Lamparski G., Amarowicz R. (2010). The effect of polysaccharides on the astringency induced by phenolic compounds. Food Qual. Prefer..

[26] Fernandes P.A., Silva A.M., Evtuguin D.V., Nunes F.M., Wessel D.F., Cardoso S.M., Coimbra M.A. (2019). The hydrophobic polysaccharides of apple pomace. Carbohydr. Polym..

[27] Wojdyło A., Jáuregui P.N.N., Carbonell-Barrachina A.A., Oszmiański J., Golis T. (2013). Variability of phytochemical properties and content of bioactive compounds in *Lonicera caerulea* L. var. *kamtschatica* berries. J. Agric. Food Chem..

[28] Rauf A., Imran M., Abu-Izneid T., Patel S., Pan X., Naz S., Silva A.S., Saeed F., Suleria H.A.R. (2019). Proanthocyanidins: A comprehensive review. Biomed. Pharmacother..

[29] Kucharska A., Sokół-Łętowska A., Oszmiański J., Piórecki N., Fecka I. (2017). Iridoids, phenolic compounds and antioxidant activity of edible honeysuckle berries (*Lonicera caerulea* var. *kamtschatica* Sevast.). Molecules.

[30] Dobler S., Petschenka G., Pankoke H. (2011). Coping with toxic plant compounds–the insect’s perspective on iridoid glycosides and cardenolides. Phytochemistry.

[31] Jurikova T., Rop O., Mlcek J., Sochor J., Balla S., Szekeres L., Hegedusova A., Hubalek J., Adam V., Kizek R. (2012). Phenolic profile of edible honeysuckle berries (genus *Lonicera*) and their biological effects. Molecules.

[32] Iwatani S., Yamamoto N. (2019). Functional food products in Japan: A review. Food Sci. Hum. Wellness.

[33] Mark R., Lyu X., Lee J.J.L., Parra-Saldívar R., Chen W.N. (2019). Sustainable production of natural phenolics for functional food applications. J. Funct. Foods.

[34] Nazir M., Arif S., Sanaullah Khan R., Nazir W., Khalid N., Maqsood S. (2019). Opportunities and challenges for functional and medicinal beverages: Current and future trends. Trends Food Sci. Technol..

[35] Kaur S., Das M. (2011). Functional foods: An overview. Food Sci. Biotechnol..

[36] Zhao C.N., Li Y., Meng X., Li S., Liu Q., Tang G.Y., Gan R.Y., Li H. (2018). Bin Insight into the roles of vitamins C and D against cancer: Myth or truth?. Cancer Lett..

[37] Zümreoglu-Karan B. (2006). The coordination chemistry of Vitamin C: An overview. Coord. Chem. Rev..

[38] Herbig A.L., Maingonnat J.F., Renard C.M.G.C. (2017). Oxygen availability in model solutions and purées during heat treatment and the impact on vitamin C degradation. LWT Food Sci. Technol..

[39] Sapei L., Hwa L. (2014). Study on the kinetics of vitamin C degradation in fresh strawberry juices. Procedia Chem..

[40] Mercali G.D., Jaeschke D.P., Tessaro I.C., Marczak L.D.F. (2012). Study of vitamin C degradation in acerola pulp during ohmic and conventional heat treatment. LWT Food Sci. Technol..

[41] Gu K.D., Wang C.K., Hu D.G., Hao Y.J. (2019). How do anthocyanins paint our horticultural products?. Sci. Hortic..

[42] Sinopoli A., Calogero G., Bartolotta A. (2019). Computational aspects of anthocyanidins and anthocyanins: A review. Food Chem..

[43] Cavalcante Braga A.R., Murador D.C., Mendes De Souza Mesquita L., Vera De Rosso V. (2018). Critical review Bioavailability of anthocyanins: Gaps in knowledge, challenges and future research. J. Food Compos. Anal..

[44] Bowen-Forbes C.S., Zhang Y., Nair M.G. (2010). Anthocyanin content, antioxidant, anti-inflammatory and anticancer properties of blackberry and raspberry fruits. J. Food Compos. Anal..

[45] Teng H., Fang T., Lin Q., Song H., Liu B., Chen L. (2017). Red raspberry and its anthocyanins: Bioactivity beyond antioxidant capacity. Trends Food Sci. Technol..

[46] Cassidy A. (2018). Berry anthocyanin intake and cardiovascular health. Mol. Aspects Med..

[47] Medina dos Santos N., Berilli Batista P., Batista Â.G., Maróstica Júnior M.R. (2019). Current evidence on cognitive improvement and neuroprotection promoted by anthocyanins. Curr. Opin. Food Sci..

[48] Kalisz S., Oszmiański J., Hładyszowski J., Mitek M. (2013). Stabilization of anthocyanin and skullcap flavone complexes–Investigations with computer simulation and experimental methods. Food Chem..

[49] Muche B.M., Speers R.A., Rupasinghe H.P.V. (2018). Storage temperature impacts on anthocyanins degradation, color changes and haze development in juice of “Merlot” and “Ruby” grapes (*Vitis vinifera*). Front. Nutr..

[50] Roidoung S., Dolan K.D., Siddiq M. (2017). Estimation of kinetic parameters of anthocyanins and color degradation in vitamin C fortified cranberry juice during storage. Food Res. Int..

[51] Buvé C., Kebede B.T., De Batselier C., Carrillo C., Pham H.T.T., Hendrickx M., Grauwet T., Van Loey A. (2018). Kinetics of colour changes in pasteurised strawberry juice during storage. J. Food Eng..

